# MicroRNA-144: A novel biological marker and potential therapeutic target in human solid cancers

**DOI:** 10.7150/jca.46293

**Published:** 2020-09-25

**Authors:** Meng Zhou, Yuncui Wu, Hongwu Li, Xiaojun Zha

**Affiliations:** 1 Department of Biochemistry & Molecular Biology, School of Basic Medicine, Anhui Medical University, Hefei 230032, China.; 2 Department of Otorhinolaryngology, Head & Neck Surgery, The Fourth Affiliated Hospital of Anhui Medical University, Hefei 230000, China.

**Keywords:** miRNA, miR-144, tumor development, cancer diagnosis, cancer prognosis, cancer treatment

## Abstract

MicroRNAs (miRNAs) are a class of small non-coding RNAs that negatively regulate gene expression at the post-transcriptional level. It has been reported that microRNA-144 (miR-144) is highly conserved and can combine complementarily with the 3'-UTRs of target gene mRNAs to inhibit mRNA translation or promote targeted mRNA degradation. MiR-144 is abnormally expressed and has been identified as a tumor suppressor in many types of solid tumors. Increasing evidence supports a crucial role for miR-144 in modulating physiopathologic processes, such as proliferation, apoptosis, invasion, migration and angiogenesis in different tumor cells. Apart from these functions, miR-144 can also affect drug sensitivity, cancer treatment and patient prognosis. In this review, we summarize the biological functions of miR-144, its direct targets and the important signal pathways through which it acts in relation to various tumors. We also discuss the role of miR-144 in tumor biology and its clinical significance in detail and offer novel insights into molecular targeting therapy for human cancers.

## Introduction

Cancer incidence and mortality are rapidly growing worldwide. In 2018, approximately 18.1 million new cancer cases and 9.6 million cancer deaths were estimated to occur throughout the world according to the most recent compilation of cancer statistics 1. In the United States, 1,806,590 new cancer cases and 606,520 cancer deaths are expected to occur in 2020 2. Moreover, cancer can lead to a tremendous burden on society in every country. Therefore, it is urgent to identify new early biomarkers and therapeutic targets for various cancers. Recently, studies have shown that microRNAs (miRNAs) can be used as promising biomarkers not only for early cancer diagnosis, but also for accurate prognosis, as well as being targets for more efficient cancer treatment.

miRNAs are short, non-protein coding RNAs approximately 21-23 nucleotides in length that regulate gene expression through binding to complementary sequences in the 3' untranslated region (UTR) of their mRNA targets, resulting in the degradation of these mRNAs or translational inhibition 3. miRNAs were originally discovered by Lee and colleagues in *Caenorhabditis elegans* in 1993 4. To date, more than 1,000 miRNAs have been identified in the human genome 5. Accumulating studies have shown that miR-144 participates in diverse biological processes, including proliferation, apoptosis, invasion, migration, cell cycle and angiogenesis. Moreover, miR-144 has been shown to have vital clinical significance in terms of drug sensitivity, treatment and patient prognosis for many cancers. In addition to cancer, miR-144 also has other biological functions, including playing roles in diabetic oxidative stress 6, cholesterol homeostasis 7, exerting cardioprotective effects 8, and involvement in Alzheimer disease (AD) 4, and the differentiation of bone mesenchymal stem cells 9. These observations, however, are not a focus of our review.

In this review, we focus on the action of miR-144 in the processes of tumor biology, such as proliferation, apoptosis, invasion, migration, cell cycle and angiogenesis, as well as its clinical significance including involvement in the treatment of cancer, patient diagnose and prognosis, and the underlying mechanisms that miR-144 acts on.

## MicroRNA-144 influences tumor biology

### The essential propertities of microRNA-144

**Figure 1:**
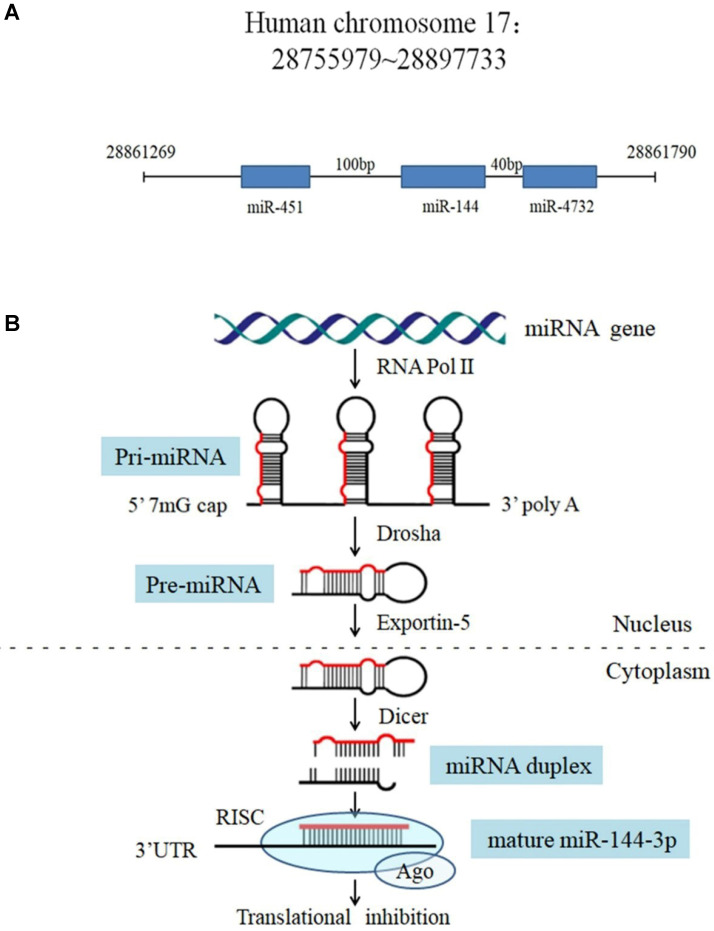
The miR-144gene locus in human genome and the processing procedure of miR-144. (**A**) Features of the miR-144 locus. (**B**) Model for miR-144 processing: RNA polymerase II transcribes the miR-144 gene to produce a pri-miRNA (with a 5' m7G cap and a 3' poly-A tail), which is processed into a pre-miR-144 by Drosha in the nucleus. Pre-miR-144 is exported to the cytoplasm and is processed into a duplex miRNA by Dicer. The passenger strand is degraded and the mature miRNA is integrated into the RISC to bind to the mRNA target.

In the human genome, the microRNA-144 (miR-144) gene is located on chromosome 17q11.2 10, 100 bp upstream of the miR-451 gene and 40 bp downstream of the miR-4732 gene (**Figure 1A**). Like other miRNAs, the biogenesis of miR-144 is a multistep process (**Figure 1B**). The miR-144 encoding gene is initially transcribed by RNA polymerase II as a long hairpin molecule (pri-miRNA) in the nucleus. This pri-miRNA is processed by an RNase III Drosha into a stem-loop-structured miRNA precursor molecule (pre-miRNA) 11. Then, pre-miR-144 is transported to the cytoplasm by the exportin-5 nucleus transporter. The cytoplasmic biogenesis process of pre-miR-144 into mature miR-144 is mediated by another human RNase III (Dicer), resulting in a miRNA duplex consisting of a mature miRNA (guide strand, miR-144-3p) and its anti-sense strand (passenger strand, miR-144-5p). Finally, the mature miRNA is bound by Argonaut (AGO) to form a miRNA-protein complex known as the RNA-induced silencing complex (RISC), while the passenger strand is degraded 12. Mounting evidence suggests that miRNAs act as oncogenes or tumor suppressors by targeting genes involved in cell differentiation, proliferation, survival, apoptosis and metastasis. Among the abundant cancer-associated miRNAs, miR-144, which can serve as a tumor suppressor, was found to be significantly dysregulated in various cancers, such as lung cancer, gastric cancer, colorectal cancer, osteosarcoma, liver cancer and thyroid cancer 13-18 (**Table 1**). Indeed, despite the well characterized role of miR-144 as an antitumor factor, there is some evidence contradictory to this role that should be mentioned in other tumor types such as nasopharyngeal carcinoma (NPC) and breast cancer where miR-144 is up regulated 19, 20.

### MicroRNA-144 in cancer proliferation and apoptosis

**Figure 2:**
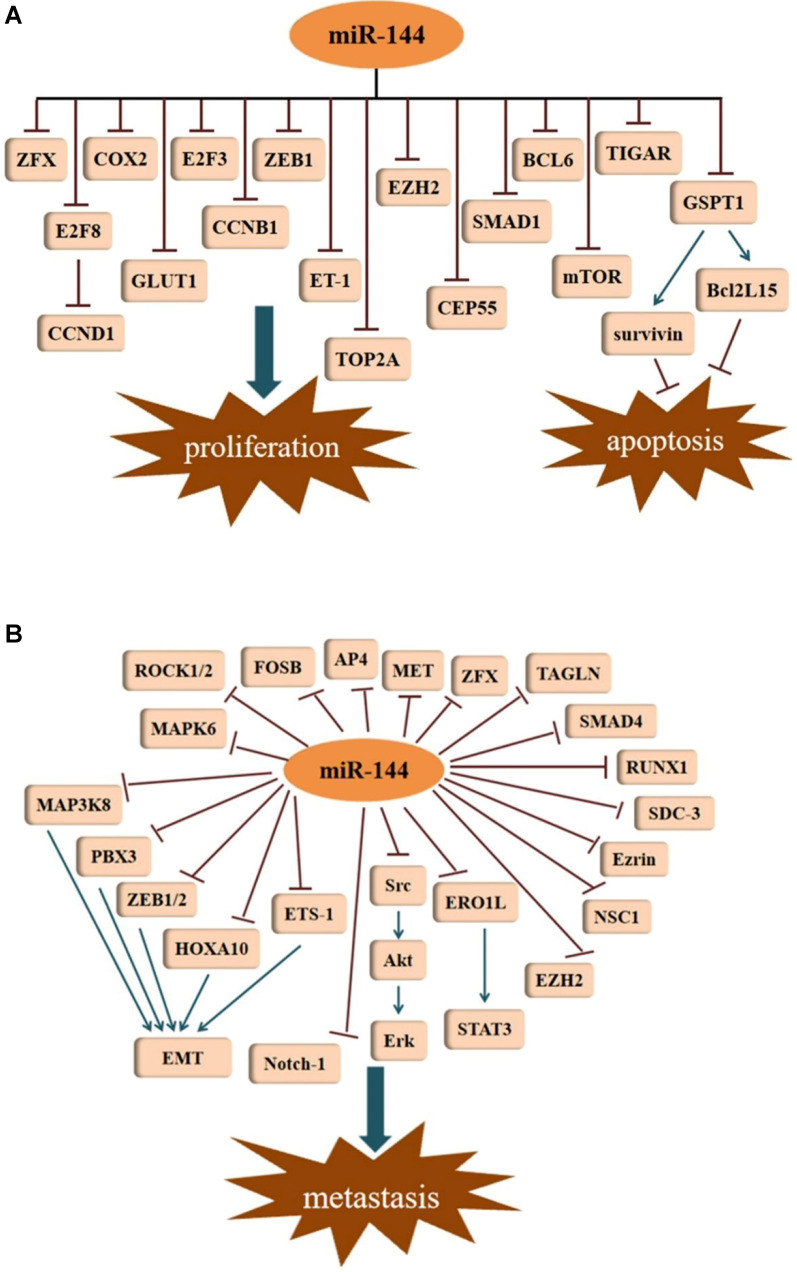
Tumor suppressive signatures of miR-144. (**A**) MiR-144 is involved in the regulation of cell proliferation and apoptosis. (**B**) MiR-144 represses metastasis by inhibiting different signal pathways.

Accelerated proliferation and decreased apoptosis in cancer cells contributes to the progression of tumor development. Studies have demonstrated that miR-144 is associated with proliferation and apoptosis in different kinds of cancer. For example, in 2015, Chen et al. 13 described that in lung cancer cells, miR-144 expression was lower. By targeting p53-induced glycolysis and apoptosis regulator (TIGAR), miR-144 was shown to inhibit proliferation, induced apoptosis, and increased autophagy in both A549 and H460 cells. Through the targeting of TIGAR, miR-144 was also shown to suppress esophageal squamous cell carcinoma (ESCC) cell proliferation 21. Likewise, evidence demonstrated an obvious down-regulation of miR-144 in non-small cell lung cancer (NSCLC) tissues and cells, which was able to inhibit cell growth as well as facilitate apoptosis through down regulation of ZFX protein expression 22. In malignant solitary pulmonary nodule (SPN) tissues and peripheral blood, the expression of miR-144 was reduced. By targeting zinc finger E-box binding homeobox 1 (ZEB1), miR-144 can limit the proliferative capacity of lung cancer cells 23. Consistent with the results, miR-144 was also shown to function as a tumor suppressor in gastric cancer (GC) by negatively regulating cyclooxygenase-2 (COX2) 24. This suppressive process of targeting COX2 was seen not only in GC cells, but also in ESCC cell lines (EC9706 and EC109) 25. Moreover, miR-144 can target Endothelin-1 (ET-1) to inhibit GC cell proliferation 26. G1 to S-phase transition 1 (GSPT1) protein may be an essential factor involved in the regulation of the proliferation and apoptosis. GSPT1 functions as an oncogene in various cancers. In GC, miR-144 suppresses cell proliferation by directly targeting GSPT1 27. GSPT1 is also responsible for the anti-proliferative effects of miR-144 in colorectal cancer (CRC). In HCT116 cells, overexpression of miR-144 results in the suppression of GSPT1, which leads to the downregulation of survivin and BCL2-like 15 (Bcl2L15) 15. Survivin and Bcl-2 family proteins exhibit their anti-apoptotic functions in tumor cells. Additionally, a recent investigation revealed that miR-144-3p could inhibit proliferation in CRC cells by targeting B-celll ymphoma 6 (BCL6) via the inhibition of Wnt/β-catenin signaling 28. Iwaya et al. 29 reported that mTOR is the direct target of miR-144 in CRC cells, and miR-144 suppression of mTOR led to the inhibition of cell proliferation. A similar observation was reported in renal cell carcinoma (RCC) 30. Moreover, miR-144-3p was markedly decreased in prostate cancer (PCa) tissues and cell lines compared with its expression in paired adjacent normal tissues and normal cell lines. Overexpression of miR-144-3p in PC-3 and DU145 cells by transfection with miR-144-3p mimics not only significantly inhibited cell proliferation *in vitro* but it also suppressed tumor growth *in vivo* via decreasing centrosomal protein of 55 (CEP55) 31. Additionally, the reduction of CEP55 may also account for the miR-144-3p inhibition of cell proliferation in castration-resistant prostate cancer (CRPC) 32. Similar results were obtained from another study, where restoration of miR-144 significantly diminished CEP55 expression in breast cancerous tissues and cells, contributed to inhibiting cancer progression *in vitro* and *in vivo* 33. In glioblastoma cell lines (U87 and U251), high expression of topoisomerase II alpha (TOP2A) promotes cell proliferation and inhibits apoptosis 34. However, miR-144-3p can directly bind to the 3'-UTR of TOP2A, and thus, decrease its expression. Overexpression of miR-144-3p can suppress proliferation in glioma cells by targeting TOP2A and inhibit the growth of glioma xenografts in nude mice. Glucose transporter isoform 1 (GLUT1) has been shown to be upregulated in many cancers, leading to an increase in glucose uptake and lactate production. Through targeting of GLUT1, miR-144 can inhibit cell proliferation in ovarian cancer cells by participating in the regulation of glucose metabolism 35. This was the first study demonstrating that miRNAs regulate glucose metabolism in ovarian cancer cells. In lung cancer, down-regulation of GLUT1 is responsible for the anti-proliferative effect induced by miR-144 36. As a member of the E2F transcription factor family, E2F transcription factor 3 (E2F3) has been shown to be an oncogene with strong proliferative potential. Through the repression of E2F3, miR-144 represses hepatocellular carcinoma (HCC) cell proliferation 17. Enhancer of zeste homolog 2 (EZH2) is an oncogenic protein implicated in multiple cancer types. In bladder cancer, down-regulation of miR-144 results in an increase of EZH2, which contributes to the activation of Wnt/β-catenin signaling and subsequent cell proliferation 37. Peng and Zhang 38 disclosed that efficient knockdown of endogenous miR-144 almost completely abrogated the proliferation inhibition of melanoma cells induced by Baohuoside-I, which indicated that miR-144 exerts a predominant role in mediating the antitumor process of Baohuoside-I for melanoma. Furthermore, SMAD1 was shown to be a novel target of miR-144 in melanoma. Nevertheless, miR-144 enhanced cell proliferation in the KYSE-410 human esophageal carcinoma cell line by targeting PURA mRNA had been proposed by R. Sharma 39 (**Figure 2A**).

### MicroRNA-144 in cancer invasion and migration

Metastasis, an important characteristic of malignant tumors, accounts for the majority of cancer-related deaths. Several studies suggest that miR-144 is a repressor of EMT. With the exception of its role in tumor growth, EZH2 also participates in the metastasis of cancers. In lung adenocarcinoma (LUAD), miR-144-3p down regulates the expression of EZH2, and thus, represses cell migration and invasion 40. As a member of the NCS family, neuronal calcium sensor 1 (NCS1) is a multifunctional protein with the ability to enhance the aggressiveness of lung cancer. Direct regulation of NCS1 by both strands of miR-144 (miR-144-5p and miR-144-3p) was shown to significantly block the migration and invasion abilities of lung squamous cell carcinoma (LUSQ) cells 41. This was the first report describing the involvement of the passenger strand miR-144-5p in LUSQ etiopathogenesis. Interestingly, miR-144-5p also serves as an antitumor factor in the migration of RCC cells by targeting syndecan-3 (SDC3) 42. SDC3 is a member of the syndecan protein family and is strongly expressed in tumor stromal vessels. Zinc finger protein, X-linked (ZFX), as a member of the highly-conserved zinc finger protein Zfy family, is intimately involved in tumor metastases. In HCC, the inhibition of ZFX by miR-144 inhibits cell invasion 43. However, miR-144-3p does not necessarily function as a tumor suppressor gene in HCC. A study showed interestingly that miR-144-3p was markedly elevated in the serum of patients with HCC 44. In GC, the suppressive role of miR-144 on cell metastasis could be attributed to the suppression of met proto-oncogene (MET) 45. The resembled a phenomenon that was also observed in uveal melanoma cells 46. In another investigation of GC, the activating enhancer-binding protein 4 (AP4) was determined to be a corresponding target of miR-144 47. Regarding osteosarcoma (OS), exogenous of miR-144 expression can restrain the invasion and migration of the OS cell line F5M2 via down-regulation of Ezrin 48. The cytosolic protein Ezrin is a member of the ERM (Ezrin, radixin, moesin) protein family that promotes cancer cell migration and invasion. Furthermore, miR-144 exerts its anti-invasive effect in OS cells through the modulation of transgelin (TAGLN) 49. Apart from Ezrin and TAGLN, the effect of miR-144 on OS cell invasion can be dependent on the inhibition of Rho-associated kinases 1 and 2 (ROCK1 and ROCK2) 16. A recent study demonstrated that miR-144 diminishes the expression of Ras homolog family member A (RhoA) and its pivotal downstream effector ROCK1, playing an anti-metastatic role in OS 50. Likewise, the downward trend seen for ROCK1 was ascribed to the anti-migration action of miR-144 in rectal carcinoma cell lines (SW837 and SW1463) 51. In ovarian cancer, upon miR-144 up-regulation, the expression of the regulator of runt-related transcription factor 1 (RUNX1) decreases, limiting invasion and migration 52. By suppressing SMAD family member 4 (SMAD4), which has been identified as a common mediator for cell motility promotion, miR-144 impedes cell migration and invasion in colon cancer 53. In laryngeal squamous cell carcinoma (LSCC) cells, an unusual expression of the miR-144, leads to the facilitated metastasis of tumor cells, as well as accelerating tumor growth *in vivo*. This is associated with the increased expression of insulin receptor substrate 1 (IRS1) in tumor cells 54. In pancreatic cancer (PC), elevated expression of miR-144-3p was able to repress cell migration and invasion by downregulating the FosB proto-oncogene, AP-1 Transcription Factor Subunit (FOSB) 55. In cervical cancer cells, MAPK6 was shown to be directly targeted by miR-144-3p to inhibit cell migration and invasion 56 (**Figure 2B**).

### MicroRNA-144 in cell cycle

The cell cycle is modulated by many complex molecular pathways. A number of miRNAs take part in these pathways. Several studies have verified that miR-144 can influence the G1-S transition in cell cycle progression to regulate tumor cell activity. The G1-S phase transition is regulated by diverse factors such as cyclin E1 (CCNE1), cyclin E2 (CCNE2), and cyclin D1 (CCND1). For instance, miR-144-5p can induce G1/S cell cycle arrest to significantly inhibit bladder cancer cell propagation through direct targeting of oncogenic genes (CCNE1, CCNE2, CDC25A, and PKMYT1) that are four cell cycle-related genes 57. Another investigation revealed that miR-144 could elevate G0/G1 phase-arrested in non-small cell lung cancer (NSCLC) cells and decrease the number S phase-arrested cells via suppression of CCNE1 and CCNE2 58. In papillary thyroid cancer (PTC), miR-144 can directly target E2F family function as transcription factor 8 (E2F8) to significantly inhibit proliferation of PTC cells by inducing G1/S arrest via down-regulating CCND1, indicating that the miR-144/E2F8/CCND1 pathway may represent a potential therapeutic strategy for PTC 59. Cyclin B1 (CCNB1) impacts the G2 to M phase transition. In HCC, miR-144 could not only suppress malignant biological behaviors in cell lines, but also inhibits tumor formation in nude mice models by negative regulation of CCNB1 60. As we will mention, miR-144-3p was shown to induce cell cycle arrest and apoptosis by targeting proline-rich protein 11 (PRR11) via affecting the mitogen-activated protein kinase (MAPK) signal pathway in pancreatic cancer 61.

### MicroRNA-144 in angiogenesis

Angiogenesis is generally accepted as a vital characteristic in various malignant neoplasms, which can significantly promote tumor growth, metastasis and even cause resistance. An increasing number of studies have revealed that miR-144 plays a crucial role in inhibiting the tumor angiogenesis. It has been reported that in animal models, up-regulation of miR‑144‑3p may suppress the angiogenic capacity of HCC cells. Meanwhile, Wu et al. 62 further demonstrated that *in vitro* and *in vivo*, miR‑144‑3p can strongly inhibit angiogenesis by targeting serum and glucocorticoid kinase 3 (SGK3), suppressing activation of mTOR/vascular endothelial growth factor receptor 2 (VEGFR2) by PI3K downstream signals. Hence, miR‑144‑3p could be a novel target for anti-angiogenic cancer therapy. Consistent with this discovery, there was a study that also reported that miR-144 can exert a suppressive effect on the growth and metastasis of Hela and C33A cells by targeting vascular endothelial growth factor A (VEGFA) and VEGFC 63. VEGFA and VEGFC, two members of the VEGF family, have been reported to act as factors that contribute to tumor angiogenesis 64.

### The cellular mechanisms of microRNA-144

Epithelial mesenchymal transition (EMT) is a process whereby epithelial cells lose their cell polarity or typical epithelial features and transform into mesenchymal cells. Cancer cells can acquire invasiveness and metastasize through the process of EMT. In malignant epithelial cells, zinc finger E-box-binding homeobox 1 and 2 (ZEB1 and ZEB2), as EMT activators, promote the phenotypic transformation of epithelial cells into mesenchymal cells. In the case of breast cancer, miR-144 limits cell migration and invasion through the reversal of EMT. Mechanistically, miR-144 reverses EMT via the suppression of ZEB1 and ZEB2 65. Similarly, the reduction of ZEB1 and ZEB2 can also account for the inhibition of cancer cell invasion mediated by miR-144 in thyroid cancer and NSCLC 66, 67. In GC cells, over-expression of miR-144 increases E-cadherin expression and reduces N-cadherin expression by down-regulating the expression of pre-leukemia transcription factor 3 (PBX3) 14. The reversal of EMT induced by miR-144 leads to the weakened invasive ability of GC cells. By up-regulating the epithelial marker E-cadherin and down-regulating the mesenchymal markers N-cadherin and vimentin, miR-144-3p reverses EMT and inhibits the migration of RCC cells. In addition, the suppression of miR-144-3p on EMT in RCC might also be attributed to the inhibition of mitogen-activated protein kinase 8 (MAP3K8) 68. Through directly targeting homeobox A10 (HOXA10), miR-144 has been shown to reverse EMT in LUAD cells, inhibiting cell migration and invasion 69.

### The molecular mechanisms of microRNA-144

MicroRNAs regulate gene expression at the post-transcriptional level by binding to the 3'-UTR of target messenger RNAs. In recent years, various studies have reported that miR-144 can regulate different genes or signaling pathways to affect tumorigenesis. miR-144 is involved in many events in tumor development and progression, such as proliferation, apoptosis, invasion, migration and angiogenesis. The molecular mechanisms whereby miR-144 can target different molecules have been demonstrated in various findings, but some specific mechanisms in tumorigenesis are still currently unclear. Apart from the EMT process, several signaling pathways are also involved in the metastasis of cancers. The aberrant activation of steroid receptor coactivator (Src) regulates the biological behaviors of several solid tumors. In lung cancer, miR-144-3p suppresses cell invasion and adhesion via regulating the Src-protein kinase B-extracellular-regulated protein kinases (Src-Akt-Erk) pathway 70. The Notch signaling pathway is frequently activated in multiple human cancers. In CRC, miR-144 represses notch-1, a member of the Notch family, and inhibits the migratory and invasive potential of cells 71. Lv et al. 72 uncovered the molecular mechanism where lncRNA taurine upregulated 1 (lncRNATUG1) functions as a molecular sponge for miR-144 to accelerate HCC tumor growth *in vitro* and *in vivo* through activation of the JAK2/STAT3 pathway. Consistent with this, miR-144 targets epidermal growth factor receptor (EGFR) and inhibits the EGFR/Src/AKT signaling axis in mouse HCC 73. In oral squamous cell carcinoma (OSCC) cells, miR-144-3p can exert a tumor suppressive function by directly targeting the ERO1L/STAT3 pathway to affect OSCC treatment 74. Conversely, research from Zhang et al. suggested that miR-144 might promote nasopharyngeal carcinoma cell migration and invasion through the repression of phosphatase and tensin homolog (PTEN) to activate the PI3K/Akt pathway 19.

### The interactions among microRNA-144 and other molecules

Various lncRNAs are associated with the regulation of miR-144 in human cancers. In recent years, the mechanism of competing endogenous RNAs (ceRNA) has attracted much attention. One study found that miR-144 may induce degradation or suppress the expression of proline rich 11 (PRR11). Moreover, lncRNA distal-less homeobox 6 antisense 1 (lncDLX6-AS1) was found to promote the expression of PRR11 in non-small cell lung cancer (NSCLC) cells by binding miR-144, and the recovery in PRR11 strengthened the proliferative ability of NSCLC cells *in vitro* 75. By directly binding to miR-144-3p, lncRNA metastasis-associated lung adenocarcinoma transcript 1 (lncMALAT1) induced the expression of ROCK1 and ROCK2, promoting metastasis in OS 76. LncRNATUG1 was also over expressed in OS. Up-regulated lncRNATUG1 acted as a sponge for miR-144-3p and exhibited carcinogenicity via an enhancement of EZH2 expression 77. Additionally, lncRNA FEZF1 antisense RNA 1 (FEZF1-AS1) also promoted OS progression, and served as a sponge for miR-144 to upregulate CXC motif chemokine receptor 4 (CXCR4) expression 78. In lung cancer, lncRNA urothelial carcinoma associated 1 (lncUCA1) sequestered miR-144, and thus, induced the expression of PBX3, a tumor promoter in numerous cancers 79. In HCC, non-coding RNA activated by DNA damage (NORAD) absorbs miR-144-3p to promote SEPT2 expression. Importantly, the mechanisms underlying HCC progression provides novel evidence for the tumor suppressive role of miR-144-3p 80. In addition, lncRNA small nucleolar RNA host gene 17 (SNHG17) functions as a ceRNA to up-regulate CD51 expression through competitively combining with miR-144 in CRPC. Up-regulation of CD51 is closely related to cancer progression and poor prognosis in multiple types of cancers 81. In LSCC, the lncRNA X-Inactive Specific Transcript (XIST) promotes the tumor progression by sequestering miR‑144 to regulate IRS1 expression 82. Li et al. found that a miR-144 mimic could prevent the GC cell proliferation induced by lncRNA FTX and ZFX was a direct regulator of miR-144, demonstrating the cross-talk among FTX, miR-144, and ZFX was critical to GC 83. In addition to lncRNAs, Qu et al. 84 reported for the first time that the circRNA hsa_circ_0020123 could interact with miR-144 in NSCLC. Functionally and mechanistically, hsa_circ_0020123 can exert oncogenic properties through the suppression of miR-144.

## Clinical significance of microRNA-144

### MicroRNA-144 as a diagnostic or prognostic biomarker

The expression level of miR-144 is different in different solid tumors, which make it a potential target for the antineoplastic agents or can directly/indirectly target genes to promote or inhibit the tumorigenesis. Emerging studies have reported that the aberrant expression of miR-144 in numerous neoplasms can be used for the diagnosis or prediction of outcomes of cancer. After examining 95 HCC samples with matching adjacent non-tumor tissues, miR-144-3p was found to correlate with disease stage in HCC, and low levels of miR-144-3p were associated with poorer outcomes 85. Takeshi and co-workers studied 280 samples from patients with RCC and found that a poor outcome was often indicated in RCC patients with down-regulated miR-144-3p 29. Apart from this, there was also strong evidence in GBM patients that low-expression level of miR-144-3p was associated with poor prognosis. Another study also revealed that miR-144-3p suppressed tumor metastasis by targeting FZD7 (Frizzled-7) in GBM cells 86. Moreover, the expression of miR-144 was found to be abnormal in the four clinical stages of cervical squamous cell carcinoma (CSCC), the main tissue types of cervical cancer, demonstrating that the expression of miR-144 has significant prognostic value for patients with CSCC 87. Furthermore, the diagnostic value of miR-144 was verified in patients by several sets of clinical evidence. For instance, one trial found significant upregulation of miR-144 in both feces and tissue samples from colorectal cancer patients and that miR-144 could be a diagnostic marker for CRC screening 88. Another trial result implied that miR-144 might be a useful indicator for CRC (*p* < 0.001, sensitivity 89.7%) 89. In the serum of oesophageal cancer patients, miR-144 expression was detected to be prominently high, and the area under the curve (AUC) was 0.731, which indicated it might be a suitable diagnostic marker 90. miR-144-3p was markedly elevated in the serum of patients with HCC and the area under the curve (AUC) was 0.780 44. Results also suggested that miR-144-3p could retain its diagnostic efficiency in HCC patients. Quantitative PCR analysis of a group of miRNAs in OSCC verified that miR-144 was significantly increased in OSCC primary tissues compared to control samples, suggesting that miR-144 might serve as a biomarker for early diagnosis of OSCC 91. The authors confirmed successively that the high expression of miR-144 in medullary thyroid carcinoma (MTC) tissue samples could be considered as a new biomarker for MTC diagnosis, but it showed no significant prognostic value as a MTC biomarker in plasma samples 92, 93. However, more investigations concerning miR-144 as a new biomarker for MTC are required since MTC is a rare cancer and there is a paucity of samples for diagnostic studies. Another controversy about miR-144 in breast cancer cell lines should be noted here 94. As the underlying mechanisms that lead to cancer development are intricate in different cell lines, caution should be taken in the selection of diagnostic or prognostic factors.

### MicroRNA-144 and cancer treatments

Both chemotherapy and radiotherapy are crucial treatments for cancer and increasing evidence has shown that the regulation of cancer sensitivity to chemotherapy or radiotherapy is affected by miR-144. Akiyoshi et al. 95 proposed that *in vitro* administration of miR-144 increases susceptibility to 5-FU by directly inhibiting ZFX in GC. Another study found that miR-144 can restore the chemosensitivity of HCC cell lines to 5-FU through partial repression of Nrf2-dependent pathway, which suggested that miR-144 might be an effective reversal agent for drug resistance in HCC in the future 96. As a target molecule of miR-144, regulation of nuclear factor erythroid-2-related factor-2 (Nrf2) affects epigenetic mechanisms 97. Moreover, Yu et al. 98 showed the SMAD4 gene was regulated by miR-144-3p and miR-144-3p could increase the chemosensitivity of 5-fluorouracil in an HCC cell line. Similar to the effect of increasing a cell's drug sensitivity, there was a study averred that the over-expression of miR-144-3p resulted in the enhancement of temozolomide (TMZ) sensitivity in GBM cells by targeting MET and then inhibiting downstream signaling 99. A similar phenomenon was observed in another study of malignant gliomas, which also confirmed miR-144 could restrict glioma progression and elevate susceptibility to TMZ by targeting CAV2 and FGF7 100. Tian and colleagues found that miR-144-3p could improve NSCLC cell line sensibility to cisplatin 101. Analogously, miR-144 over-expression may be a promising therapeutic strategy to overcome GBM cell invasiveness and resistance to chemotherapy 102. Moreover, miR-144 in combination with dichloroacetate combined therapy could prevent the drug resistance of glioma cells, therefore deserving of further exploration in the future. A new direction for the treatment of cholangiocarcinoma (CCA) was addressed by Yang et al. where miR-144 could induce resistance to chemotherapy via the miR-144/LIS1/AKT pathway 103. In cervical cancer cells, miR-144 reversed resistance to cisplatin via promoting cell apoptosis and inhibiting invasion through targeting of LIM homeobox 2 (LHX2) 104. In addition to all of that, enhancing miR-144 expression could remarkably sensitize anaplastic thyroid carcinoma (ATC) cells to cisplatin through suppressing transforming growth factor (TGF)-α 105. TGF-α is an epidermal growth factor (EGF)-related protein, and is highly expressed in most kinds of thyroid carcinomas. In contrast, another study found that miR-144-3p weakened the sensitivity of PTC cells to paclitaxel because there was a negative relationship between miR-144-3p and paired box gene 8 (PAX8) 20. With regard to radiosensitivity, it was reported that miR-144 could enhance radiosensitivity by targeting HOXA10 in a lung cancer cell line (LTEP-A-2) 69. In another study, the lncTUG1/miR-144-3p axis was shown to affect the radiosensitivity of ESCC cells 106. Moreover, there was also a study that reported that miR-144-5p increased the sensitivity of NSCLC to radiation therapy by targeting activating transcription factor 2 (ATF2), implying that miR-144-5p could be a biomarker for radiation therapy response 107. Except for the inhibitory effects on resistance to radiation therapy, miR-144 was also reported have an opposite effect in breast cancer. There was a study that showed that both MDA-MB-231 and SKBR3 cells exhibited significantly increased radiation resistance after over-expression of miR-144 108. Therefore, miR-144 plays an indispensable role in the therapy of cancer and further experiments utilizing miR-144 to treat cancer are urgent.

## Conclusions

In this review, we focused on the function of miR-144 in tumor molecular biology and its clinical significance in various human solid tumors. It can play a vital role in the progression of many cancers. In tumor molecular biology, miR-144 exerts a tumor suppressive effect and influences different tumor cellular processes, such as proliferation, apoptosis, invasion, migration and angiogenesis. In terms of clinical significance, miR-144 can affect clinical therapy, drug sensitivity in patients and a patients' prognosis. In detail, miR-144 regulates various target genes to inhibit tumor growth, such as GSPT1, EZH2, Notch-1, mTOR, etc. 15, 29, 40, 71. A large body of recent evidence has suggested that miR-144 participates in tumor biology and could be an excellent candidate for clinical treatment. Current studies of miR-144 function are limited to cellular and *in vitro* xenograft experiments but a miR-144 gene knockout mouse model is necessary for future studies. At the same time, the regulatory mechanisms of miRNA molecules are extremely complex and variable, as we have found that miR-144 has multiple target genes and complex signaling pathways when it plays a role in the same or different cancers. As such, further study is needed to explore the relationships and interactions between miR-144 and cancers, as well as relevant signaling pathways and biological processes, which may lead to new diagnostic and therapeutic approaches. However, there are still some questions to answer with regards to miR-144. How many downstream targets of miR-144 are there? What should be the therapeutic application of miR-144 and its targets? The mechanism of action of miR-144-3p and miR-144-5p requires significant further exploration, especially in terms of the untapped potential of miR-144-5p. Aside from the well-documented tumor inhibitory effect of miR-144, the carcinogenic effect of miR-144 also still needs further validation. Although some articles 19, 20, 108 have mentioned the carcinogenic effect of miR-144, it exerts an antitumor effect in the majority of studies, which overwhelmingly indicates that miR-144 serves as a tumor suppressor in most solid tumors. In conclusion, we have highlighted a significant portion of the literature related to miR-144 to help promote future research, which is clinically relevant.

## Figures and Tables

**Figure 3:**
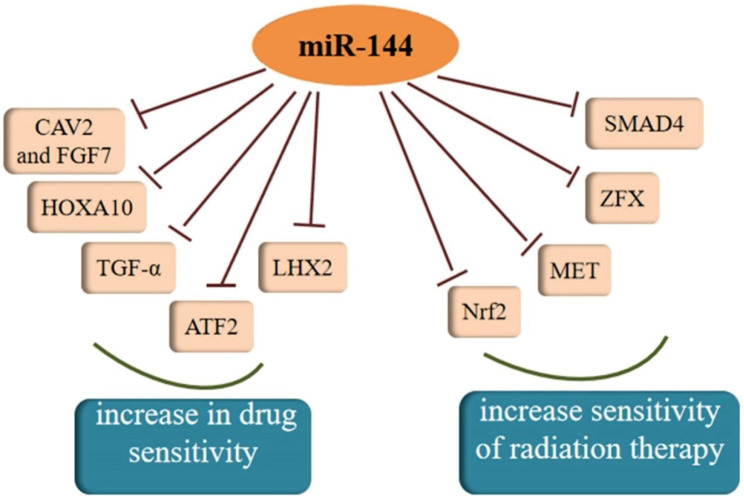
MiR-144 was confirmed to increase sensitivity of drug and radiation therapy, which could be a target for cancer treatment.

**Table 1:** Function and direct target genes of miR-144 in cancers

Gene	Function	Cancer types	Targets	Experimental model	First author reference
miR-144	antitumor	lung cancer	TIGAR	cellular system animal model	Chen 13
miR-144	antitumor		ZFX	cellular system	Zha 22
miR-144	antitumor		ZEB1	cellular system	Zhang 23
miR-144	antitumor		GLUT1	cellular system	Liu 36
miR-144	antitumor		EZH2	cellular system animal model	Liu 40
miR-144-3pmiR-144-5p	antitumor		NCS1	cellular system	Uchida 41
miR-144	antitumor		CCNE1/CCNE2	cellular system animal model	Liang 58
miR-144	antitumor		ZEB1 and ZEB2	cellular system animal model	Pan 66
miR-144	antitumor		HOXA10	cellular system animal model	Yang 69
miR-144	antitumor		Src	cellular system	Jiang 70
miR-144	antitumor		PRR11	cellular system animal model	Huang 75
miR-144	antitumor		PBX3	cellular system	Li 79
miR-144-5p	antitumor		ATF2	cellular system animal model	Song 107
miR-144	antitumor	gastric cancer	COX2	cellular system	Yao 24
miR-144	antitumor		ET-1	cellular system	Tsai 26
miR-144	antitumor		GSPT1	cellular system	Tian 27
miR-144	antitumor		MET	cellular system	Liu 45
miR-144	antitumor		AP4	cellular system	Mushtaq 47
miR-144-3p	antitumor		PBX3	cellular system	Li 14
miR-144	antitumor		ZFX	cellular system animal model	Li 83
miR-144	antitumor		ZFX	cellular system	Akiyoshi 95
miR-144	antitumor	CRC	GSPT1	cellular system	Xiao 15
miR-144-3p	antitumor		BCL6	cellular system	Sun 28
miR-144	antitumor		mTOR	cellular system	Iwaya 29
miR-144	antitumor		ROCK-1	cellular system	Cai 51
miR-144	antitumor		SMAD4	cellular system	Sheng 53
miR-144	antitumor		Notch-1	cellular system animal model	Sureban 71
miR-144	antitumor	OS	Ezrin	cellular system	Cui 48
miR-144	antitumor		TAGLN	cellular system animal model	Zhao 49
miR-144	antitumor	OS	ROCK1 and ROCK2	cellular system animal model	Wang 16
miR-144	antitumor		RhoA	cellular system animal model	Liu 50
miR-144-3p	antitumor		EZH2	cellular system	Cao 75
miR-144	antitumor		CXCR4	cellular system	Liu 78
miR-144	antitumor	HCC	E2F3	cellular system	Cao 17
miR-144	antitumor		ZFX	cellular system	Bao 43
miR-144	antitumor		CCNB1	cellular system animal model	Gu 60
miR-144	antitumor		SGK3	cellular system animal model	Wu 62
miR-144	antitumor		JAK2	cellular system	Lv 72
miR-144	antitumor		EGFR	cellular system animal model	He 73
miR-144-3p	antitumor		SEPT2	cellular system animal model	Tian 80
miR-144	antitumor		Nrf2	cellular system	Zhou 96
miR-144-3p	antitumor		SMAD4	cellular system	Yu 98
miR-144	antitumor	thyroid cancer	E2F8	cellular system animal model	Sun 59
miR-144	antitumor		ZEB1 and ZEB2	cellular system	Guan 67
miR-144	antitumor		TGF-α	cellular system animal model	Liu 105
miR-144-3p	antitumor	GBM	TOP2A	cellular system animal model	Song 34
miR-144-3p	antitumor		FZD7	cellular system	Cheng 86
miR-144-3p	antitumor		MET	cellular system	Lan 96
miR-144	antitumor		CAV2 and FGF7	cellular system animal model	Liu 100
miR-144	antitumor	RCC	mTOR	cellular system	Xiang 30
miR-144-5p	antitumor		SDC3	cellular system	Yamada 42
miR-144-3p	antitumor		MAP3K8	cellular system	Liu 68
miR-144-3p	antitumor	cervical cancer	MAPK6	cellular system animal model	Wu 56
miR-144	antitumor		VEGFA and VEGFC	cellular system	Tao 63
miR-144	antitumor		LHX2	cellular system	Shi 104
miR-144-3p	antitumor	prostate cancer	CEP55	cellular system animal model	Zheng 31
miR-144-3p	antitumor		CEP55	cellular system	You 32
miR-144	antitumor		CD51	cellular system animal model	Bai 81
miR-144	antitumor	bladder cancer	EZH2	cellular system	Guo 37
miR-144-5p	antitumor		CCNE1/2	cellular system	Matsushita 57
miR-144	antitumor	ovarian cancer	Glut1	cellular system	Fan 35
miR-144	antitumor		RUNX1	cellular system	Han 52
miR-144	antitumor	ESCC	TIGAR	cellular system animal model	Mu 21
miR-144	antitumor		COX2	cellular system animal model	Shao 25
miR-144-3p	antitumor		MET	cellular system animal model	Wang 103
miR-144	antitumor	LSCC	IRS1	cellular system animal model	Wu 54
miR-144	antitumor		IRS1	cellular system animal model	Cui 82
miR-144-3p	antitumor	OSCC	ERO1L	cellular system animal model	Li 74
miR-144	antitumor	CCA	LIS1	cellular system animal model	Yang 103
miR-144	antitumor	breast cancer	CEP55	cellular system animal model	Yin 33
miR-144	antitumor		ZEB1/2	cellular system	Pan 65
miR-144	antitumor	melanoma	SMAD1	cellular system animal model	Peng 38
miR-144	antitumor	uveal melanoma	MET	cellular system	Sun 46
miR-144-3p	antitumor	pancreatic cancer	FOSB	cellular system	Liu 55

CRC, colorectal cancer; OS, osteosarcoma; HCC, hepatocellular carcinoma; GBM, glioblastoma; RCC, renal cell carcinoma; ESCC, esophageal cancer; LSCC, laryngeal squamous; OSCC, oral squamous cell carcinoma; CCA, cholangiocarcinoma.
